# Aromatic amino acid metabolism and active transport regulation are implicated in microbial persistence in fractured shale reservoirs

**DOI:** 10.1093/ismeco/ycae149

**Published:** 2024-11-26

**Authors:** Chika Jude Ugwuodo, Fabrizio Colosimo, Jishnu Adhikari, Samuel O Purvine, Elizabeth K Eder, David W Hoyt, Stephanie A Wright, Mary S Lipton, Paula J Mouser

**Affiliations:** Natural Resources and Earth Systems Science, University of New Hampshire, Durham, NH 03824, United States; Department of Civil and Environmental Engineering, University of New Hampshire, Durham, NH 03824, United States; New England Biolabs, Ipswich, MA, 01938, United States; Tetra Tech Inc., King of Prussia, PA, 19406, United States; Environmental and Biological Sciences Directorate, Pacific Northwest National Laboratory, Richland, WA, 99352, United States; Environmental and Biological Sciences Directorate, Pacific Northwest National Laboratory, Richland, WA, 99352, United States; Environmental and Biological Sciences Directorate, Pacific Northwest National Laboratory, Richland, WA, 99352, United States; Environmental and Biological Sciences Directorate, Pacific Northwest National Laboratory, Richland, WA, 99352, United States; Environmental and Biological Sciences Directorate, Pacific Northwest National Laboratory, Richland, WA, 99352, United States; Department of Civil and Environmental Engineering, University of New Hampshire, Durham, NH 03824, United States

**Keywords:** proteomics, *Halanaerobium*fractured shale, salinity, hydraulic retention time, metabolomics, produced water

## Abstract

Hydraulic fracturing has unlocked vast amounts of hydrocarbons trapped within unconventional shale formations. This large-scale engineering approach inadvertently introduces microorganisms into the hydrocarbon reservoir, allowing them to inhabit a new physical space and thrive in the unique biogeochemical resources present in the environment. Advancing our fundamental understanding of microbial growth and physiology in this extreme subsurface environment is critical to improving biofouling control efficacy and maximizing opportunities for beneficial natural resource exploitation. Here, we used metaproteomics and exometabolomics to investigate the biochemical mechanisms underpinning the adaptation of model bacterium *Halanaerobium congolense* WG10 and mixed microbial consortia enriched from shale-produced fluids to hypersalinity and very low reservoir flow rates (metabolic stress). We also queried the metabolic foundation for biofilm formation in this system, a major impediment to subsurface energy exploration. For the first time, we report that *H. congolense* WG10 accumulates tyrosine for osmoprotection, an indication of the flexible robustness of stress tolerance that enables its long-term persistence in fractured shale environments. We also identified aromatic amino acid synthesis and cell wall maintenance as critical to biofilm formation. Finally, regulation of transmembrane transport is key to metabolic stress adaptation in shale bacteria under very low well flow rates. These results provide unique insights that enable better management of hydraulically fractured shale systems, for more efficient and sustainable energy extraction.

## Introduction

Over the past few decades, hydraulic fracturing, combined with horizontal drilling, has helped the energy industry unlock vast amounts of natural gas trapped beneath unconventional formations, especially black shale [1, 2]. This process involves high pressure injection of a water-based fluid, containing proppants and several chemical additives, downhole to extend fracture networks in the underlying rock. Later in the process, flowback and produced water (FPW) are co-collected with natural gas at the well surface. During fracking, microbes are inadvertently introduced into this hostile subsurface environment characterized by very high temperatures and pressures, nutrient limitation, brine-level salinities, and anoxia [2–6]. Many of them, predominantly halotolerant anaerobic bacteria, adapt and persist. *Halanaerobium congolense* is very commonly found in global hydrocarbon reservoirs including fractured shale [7–9], and is hence a model organism for anaerobic, halophilic microorganisms that can dominate engineered subsurface shale. However, other genera have been found to be more dominant than *Halanaerobium* in relatively lower salinity shale formations including the Bowland shale in the UK [10] as well as reservoirs in the Denver-Julesburg [11] Anadarko [3] and Michigan [12] Basins. Many persistent microbes in shale are undesirable because of diverse biofouling potential, including reservoir souring with hydrogen sulfide [13], secretion of corrosive chemicals [13, 14], and clogging of hydrocarbon flow networks with biofilms (aggregates) [14]. However, some can generate methane which enhances secondary natural gas recovery [5, 15]. Therefore, advancing our limited knowledge of engineered shale microbiology is important for effective biofouling control and harnessing their beneficial potential toward more efficient and sustainable energy extraction.

Multiple studies have elucidated aspects of the microbial ecology of fractured shale reservoirs including community composition and succession, growth kinetics, nutrient cycling, stress adaptation, and biofilm formation [2, 3, 5, 13, 16]. Hydraulic fracturing fluids, rich in nutrients, provide the initial chemistry for microbial life to thrive [5]. Certain halotolerant/halophilic and anaerobic bacterial and archaeal taxa have been found to dominate the microbiome of high salinity shale reservoirs at later time points post-fracturing, including *Halanaerobium, Marinobacter*, Halomonadaceae, *Methanohalophilus*, and *Methanolobus* [5]. These microbes oxidize and/or ferment several available carbon substrates, including sucrose, ethylene glycol, and glycine betaine (GB), and excrete waste products that include gases, organic acids, and biomethane [5, 13, 16]. Some members of the persistent genera use sulfate and/or thiosulfate as oxidative electron acceptors, resulting in the production of hydrogen sulfide, a highly undesirable sour gas which also facilitates corrosion of engineering infrastructure [13]. Moreover, an intricate web of biotic interactions, including bacterial-archaeal co-metabolism and viral predation, is definitive of this system [3, 5, 16]. Some taxa, like *Halanaerobium*, ferment GB which generates trimethylamine (TMA) that is then consumed by methanogens (e.g. *Methanohalophilus*) to produce methane [5]. In addition, viral genomes have been linked to several bacterial taxa in this system. Viral predation could mobilize key nutrients, and therefore drive community evolution and biogeochemical cycling [3].

Hypersalinity and variable natural gas/produced fluid recovery flow rates (inversely related to hydraulic retention time [HRT]) are two key perturbants of the shale reservoir microbiome. HRT is the length of time input fluids spend underground in shale reservoirs (or within a laboratory chemostat) before recovery. It fluctuates during well production affecting system biogeochemical parameters such as nutrient flux and, therefore, is a determinant of microbial survival, community structure, and functions. Cells are metabolically stressed under very high HRT/low flow rate. Microbial coping strategies for high salinity and HRT remain poorly understood in this system.

Borton and co-workers established that shale bacteria use both the salt-in and osmolyte strategies to cope with hypersalinity [16], which is characteristic of engineered rock formations. Salt-in strategy involves cellular uptake of salt ions such as Na^+^ and K^+^ to increase cytosolic osmotic potential. The osmolyte strategy achieves a similar outcome, through osmoprotectant accumulation via extracellular acquisition or de novo synthesis. This current study seeks to further advance our understanding of the complexity of hypersalinity tolerance strategies used by persistent shale bacteria. Moreover, no previous study has investigated the underlying biochemistries of metabolic stress tolerance stemming from flow rate fluctuations and biofilm formation which are integral to long-term persistence. This investigation fills this very crucial knowledge gap.

All microbial phenotypes and functions including basic metabolism and response to adversities, are subject to molecular controls. These include gene expression regulation and pathway modulation. Biochemical pathways comprise interlinked networks of sequential substrate and metabolite turnovers, catalyzed by enzymes. Hence, proteomics and metabolomics, which assay cellular proteomes and metabolomes, respectively, have become key to advancing our understanding of microbial physiologies and community ecology across environments [17–21].

In light of this, we integrate (meta)proteomics with exometabolomics to investigate the biochemical mechanisms underlying the adaptation of *H. congolense* WG10 and mixed microbial consortia enriched from shale-produced fluid to nonoptimal gradients of salinity and HRT. We hypothesize that proteins and pathways involved in both salt-in and osmolyte accumulation strategies will be enriched under high salinity compared to the optimum, and also, metabolic stress-response indicators and coping mechanisms will be upregulated under high HRT. We also probe the molecular basis of biofilm formation. Finally, we discuss implications of our results for shale microbiology management and suggest data-driven measures to improve the efficiency of energy extraction from fracturing wells.

## Materials and methods

### Field sampling

Samples of produced fluids were collected from the gas-water separator of hydraulically fractured MSEEL II Boggess shale wells in the Appalachian Basin, West Virginia (approximate coordinates: 39°39′59.2″N, 80°05′48.8″W), on four occasions—December 2019, July 2020, December 2020, and May 2021. These wells started production in November 2019. Portions of the raw fluids were stored in 1 L sterile amber glass containers and the remaining fractions (10 l) were filtered using 0.22 μm mixed cellulose ester filters of diameter 90 mm (Millipore-Sigma, Burlington, MA, USA) and 0.45 μm polyethersulfone (PES) filters (EMD Millipore, Burlington, MA, USA). The filtrates were retained in the collecting sterile glass containers and the 0.22 μm filters containing microbial residues were placed in sterile Ziploc® bags. All samples were transported on dry ice back to the laboratory and stored at −80°C until analysis.

### Planktonic growth experiments

We obtained *H. congolense* WG10 (NCBI Assembly accession number: GCA_900102605.1) previously isolated from produced fluids collected from a natural gas well in Ohio, USA [13]. This strain was cultivated at 40°C in chemostat bioreactors (Sartorius Biostat Q-plus, Göttingen, Germany) containing a defined saltwater liquid medium comprising NH_4_Cl (1 g l^−1^), MgCl_2_.6H_2_O (10 g l^−1^), CaCl_2_.2H_2_O (0.1 g l^−1^), KCl (1 g l^−1^), cysteine (0.5 g l^−1^), Wolfe trace element solution (10 g l^−1^), and Wolfe vitamin solution (10 g l^−1^), and amended with D-Glucose (10 mM), NaNHCO_3_ solution (0.2%), and 0.003% phosphate solution (KH_2_PO_4_ and K_2_HPO_4_) after autoclaving. Sodium chloride was added to the medium at three concentrations—70 g l^−1^ (7%), 130 g l^−1^ (13%), and 200 g l^−1^ (20%). The salinity of produced fluids from fractured shale typically ranges from 40 000 (4%) to 280 000 (28%) mg l^−1^ [22] but could be higher depending on formation geology. NaCl is a relevant salt species because it is usually present in the water used to compose the fracturing fluid, at levels depending on source, and is also released into the reservoir fluids through processes such as halite dissolution [23]. The reactor was operated under three HRTs—19.2, 24, and 48 h. In another setup, unfiltered produced fluids were enriched at 40°C in similar reactors but with no added nutrients, under three HRTs (19.2, 48, and 72 h). Salinity was not manipulated for this experimental setup. For both the pure isolate and mixed consortia setups, the vessels were maintained in anaerobic conditions (bioreactor was equipped with an oxygen electrode, enabling online monitoring) by constantly bubbling a mixture of high-purity N_2_ and CO_2_ (80:20) into it and pH was maintained at 7.0 through automatic addition of 1 M HCl and 1 M NaOH. The density (OD600nm) of the medium was monitored daily until steady state was attained (5–8 days). Cells were then recovered by centrifugation in 50 ml tubes at 4000 × *g* for 30 min and preserved at −80°C prior to transport to the Pacific Northwest National Laboratory (PNNL), Washington, USA, on dry ice.

### Biofilm growth experiments

*Halanaerobium congolense* WG10 was incubated in a continuous drip flow biofilm reactor (Biosurface Technologies, Bozeman, MT, USA) designed for growth under low shear conditions. The reactor had six parallel test channels each supporting a silica coupon (75 mm × 25 mm × 1 mm). After inoculation with culture growing in the exponential phase, the system was fed with defined saltwater liquid medium of three salinities (7%, 13%, and 20% NaCl) amended with 25 mM glucose after autoclaving, at the constant rate of 0.35 ml min^−1^. In a separate setup, mixed microbial consortia in shale-produced fluids were enriched in a similar biofilm reactor with no nutrient amendment. The duration of incubation for both setups was 48 h. Afterward, coupons containing biofilms were extracted from the reactor and immersed in 10 ml phosphate-buffered saline. It was vortexed to dislodge the biofilm into the solution. The coupon was removed, and the suspension was centrifuged to recover the cell pellet, which was then frozen at 80°C and transported to PNNL on dry ice.

### Protein extraction from cell pellets

Cellular proteins were extracted from *H. congolense* WG10 and mixed microbial consortia (enrichment cultures and filters) using the procedure described by Nicora *et al*. [24]. Briefly, cultures and filters were suspended in ice-cold 2:1 chloroform:methanol (v/v) and vortexed for 10 min at 4°C. Samples were placed inside a −80°C freezer for 15 min to completely cool them down. Then they were centrifuged at 4000 × *g* for 5 min to separate the layers. After the upper metabolite layer was removed, the protein interlayer was recovered and placed in a new tube. Ice-cold methanol was added to the tube containing the protein interlayer, which was then vortexed and centrifuged at 4000 × *g* for 5 min. The protein interlayer was frozen in liquid nitrogen and dried in a lyophilizer for 2 h. Protein solubilization buffer, comprising 4% sodium dodecyl sulfate (SDS) and 100 mM dithiothreitol (DTT) in 50 mM tris buffer, pH 8.0, was added to the tube containing the protein pellet. It was sonicated and placed in a tube rotator for 30 min at 300 rpm. Afterward, the sample was centrifuged at 4500 × *g* for 10 min to recover the supernatant. The solubilization step was repeated one time, with both supernatants pooled together. This mixture was centrifuged at 8000 × *g* for 10 min in a fixed angle bucket rotor. The resulting supernatant was decanted into a clean tube, to which 7.5 ml of trichloroacetic acid was added. After overnight incubation at −20°C, protein precipitates were pelleted by centrifugation at 4500 × *g* for 10 min. They were washed twice with ice-cold 100% acetone and dried in a fume hood, then resuspended in protein solubilization buffer, and vortexed into solution. This mixture was shaken in an incubator/shaker for 30 min at 300 rpm to achieve complete solubilization. Proteins were digested using Filter-Aided-Sample-Preparation kits according to the manufacturer’s instructions. The resulting peptides were dried and diluted for mass spectrometry analysis.

### Proteomic mass spectrometry data acquisition and analysis

Mass spectrometry data were acquired using a Q-Exactive Plus (Thermo Scientific, Waltham, MA, USA) connected to a nanoACQUITY UPLC (Ultrahigh Pressure Liquid Chromatography) M-Class liquid chromatography system (Waters, Milford, MA, USA). Briefly, peptides were resolved on a 70-cm column packed with Phenomenex Jupiter 3 μm C18 particles at a flow rate of 0.5 ml min^−1^, using two mobile phases—A: 10 mM ammonium formate, pH 10.0, and B: 10 mM ammonium formate, pH 10.0, in acetonitrile (10:90). Elution gradients were progressively adjusted over 95 min from 100% to 30% A. The MS/MS spectra of eluting peptides were acquired and searched against reference databases using MSGF+ [25]. Commonly observed contaminants including trypsin, keratin, and albumin, with 16 entries, were included in the collections searched. A parent mass error tolerance of ±20 ppm was allowed. Also, parent signal isotope correction, variable oxidation of methionine, and partial tryptic enzyme cleavage rules were implemented. False discovery rate (FDR) was controlled using the target-decoy strategy [26]. An in-house program was used to collate the data, which was then imported into an SQL server database, filtered to ~1% FDR and combined at the protein level to provide unique peptide and spectral counts. Normalized spectral abundance frequency calculations were used to normalize spectral count data for each identified protein, based on protein length and number of proteins per sample.

### Metabolomic nuclear magnetic resonance data acquisition and analysis

Exometabolomic analysis of samples was done by nuclear magnetic resonance (NMR) at the Pacific Northwest National Laboratory. Cell-free growth medium was diluted 10% by volume with 5 mM internal standard 2,2-dimethyl-2-silapentane-5-sulfonate-*d_6_* (DSS-*d6*) and transferred to a 3-mm NMR tube for analysis. NMR spectra were acquired on a Bruker Neo spectrometer operating at 18.8 T (1H ν0 of 800.30 MHz) equipped with a 5-mm Bruker inverse triple resonance cryoprobe, hydrogen-carbon-nitrogen (TCI/CP HCN), with Z-gradient at a regulated temperature of 298.0 K. Prior to acquiring measurements, the 90° 1H pulse was calibrated. Chemical shifts were referenced to the 1H or 13C methyl signal in DSS-*d6* at 0 ppm. A pre-saturation pulse was applied to water for 1.5 s before one-dimensional ^1^H spectra, with 2048 transients at 20 ppm spectral width, was acquired using the Bruker noesypr1d pulse sequence. At a 100 ms NOE mixing time, spectral acquisition lasted 4 s. From 57 472 total points, the time domain free induction decays were zero-filled to 131 072 prior to Fourier transformation. To determine the identity of candidate metabolites, we matched the chemical shift, J-coupling, and intensity information of experimental NMR signals against reference NMR signals in the Chenomx, Human Metabolome Database, and custom in-house databases using the Chenomx NMR Suite 8.3 with quantitation relative to the internal standard. We supported 1D ^1^H metabolite assignments by acquiring 2D spectra on most of the samples, using ^1^H-^13^C heteronuclear single-quantum correlation spectroscopy and ^1^H-^1^H total correlation spectroscopy. MestReNova 14 was used to measure signal-to-noise ratios (SNR) with the limits of quantification and detection set to an SNR of 10 and 3, respectively. MestReNova 14 was also used to process TOCSY and HSQC spectra and identify ^1^H and ^13^C chemical shifts.

### Bioinformatics and statistics

Pairwise differential analysis between sample treatments was performed using both proteomic and exometabolomic data. Differential abundance was assessed based on effect size (log2 fold change [FC]) and significance measure (*P*-value) from two-tailed Student’s *t*-test. Both statistics were computed in Microsoft Excel. This was displayed using a volcano plot where molecules in the upper right and upper left quadrants are differentially upregulated and downregulated, respectively, relative to the indicated direction of comparison. Volcano plots were generated in RStudio running on R version 4.4.1 using the following packages: ggplot2, ggrepel, dplyr, and viridis. The ggplot2 functions *ggplot* and *geom_point* (the parameter *aes* was used to define color, size, and shape) were used to produce the base scatter plot; *scale_color_manual* was used to manually define the color coding, *geom_text_repel* (the parameter *data* was set to a subset of differential proteins) was used to label the points; and *geom_hline* & *geom_vline* were used to draw the y- and x intercepts, respectively. Functional enrichment analysis was done using the Database for Annotation, Visualization and Integrated Discovery (DAVID) v6.8 [27, 28]. UniProtKB IDs of differentially expressed proteins (DEPs) were entered and searched against a background of *H. congolense* proteome in the DAVID database. This process outputs cellular compartments, biological processes, and molecular functions enriched in cells under a given treatment condition. The significance of enrichment is determined based on the number of proteins in the cluster and *P*-value corrected by the Benjamini–Hochberg method for multiple testing.

## Results and discussion

### Enrichment of “salt-in” proteins and tyrosine accumulation are observed in *H. Congolense* WG10 under high salt conditions

**Figure 1:**
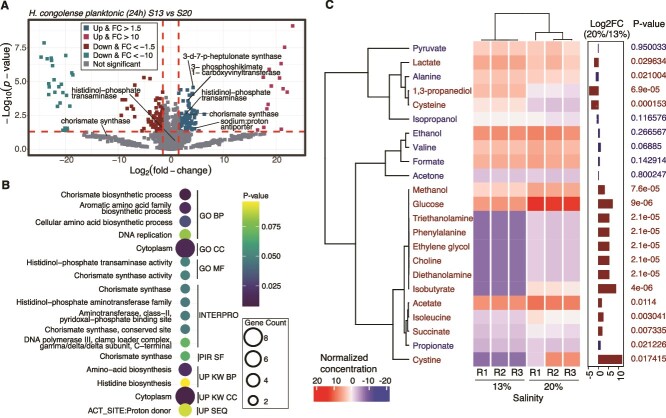
Protein and exometabolome changes in *H. congolense* WG10 grown under 13% (optimum) vs. 20% (high) NaCl. (A) Volcano plot showing the *P*-value and log2 FC of proteins detected in *H. congolense* WG10 planktonic cells grown at 24 h HRT under 13% vs. 20% NaCl (*N* = 3). The direction of comparison is 13% to 20%. The dotted horizontal line indicates the threshold of statistical significance, *P* < .05, while both dotted vertical lines delineate |FC| > 1.5. (B) Functional enrichment of discriminant (FC > 1 and *P* < .05) proteins in *H. congolense* WG10 planktonic cells upregulated under 20% NaCl. Bubble size indicates gene count. GO BP, gene ontology biological process; GO CC, gene ontology cellular compartment; GO MF, gene ontology molecular function; PIR SF, protein information resource superfamily; UP KW BP, UniProt keywords biological process; UP KW CC, UniProt keywords cellular compartment; UP SEQ, UniProt sequences. (C) Heatmap of normalized and scaled concentrations of extracellular metabolites produced by *H. congolense* WG10 planktonic cells grown at 24 h HRT, under 13% vs. 20% NaCl, annotated with a bar plot showing the log2 FC and corresponding *P*-values (*N* = 3). Statistical significance is defined as |FC| > 1.5 and *P* < .05. Rows and columns are clustered by Pearson’s correlation.

We assessed differences in the whole cell proteomes of *H. congolense* WG10 grown under optimal salinity (13% NaCl) vs. hypersalinity (20% NaCl), in both planktonic (incubated at the experimentally-determined optimum HRT, 24 h) and biofilm (HRT not applicable) modes. Based on predetermined effect size (|log2 FC| > 1.5) and statistical (*P* < .05) thresholds, 193 proteins were found to significantly differ in planktonic cells grown under 13% vs. 20% salinity (Fig. 1A). Of these 193 differential proteins, 87 (45%) were increased under 20% NaCl. On the other hand, we identified only 42 differential proteins in biofilm cultures growing under 13% vs. 20% NaCl. Only 11 out of these increased under 20% NaCl (SI Appendix, Fig. 1A). Expectedly, *H. congolense* WG10 biofilm cells are more resilient to salinity spikes than their planktonic counterpart, based on the substantially less proteomic adjustments observed (Fig. S1A). For subsequent functional analysis, we incorporated other significantly different (*P* < .05) proteins whose |FC| was less than 1.5 but greater than zero.

Several of the proteins upregulated in *H. congolense* WG10 planktonic cells under high salinity compared to the optimum were transmembrane transporters, including ATP-binding cassette (ABC) transporters and the sodium: proton antiporter. This indicates increased solute and metabolite transport activity across the membrane bilayer. An increase in the abundance of the sodium: proton antiporter is direct evidence that *H. congolense* uses the “salt-in” strategy to cope with high salinity (osmotic) stress. This transporter mediates the exchange of Na^+^ and H^+^ between the cell and its surroundings to control intracellular ionic damage and establish isotonic equilibrium. The “salt-in” strategy is a well-known salinity tolerance mechanism in bacteria [29, 30]. It has been previously shown to be the predominant osmoprotection mechanism in several shale microbial taxa including *Halanaerobium* [3, 5, 16].

We did not observe a similar increase in abundance of the sodium:proton antiporter in *H. congolense* WG10 biofilm cells growing under high salinity vs. the optimum. It is possible that *H. congolense* WG10 biofilm cells may rely on other ways to tolerate salt due to certain metabolic, mechanistic, or physiological constraints, instead of using the salt-in approach. For example, we have previously observed that zwitterionic lipids predominantly increase in *H. congolense* WG10’s plasma membrane during biofilm formation [31]. This increase reduces the negative charge of the cell envelope, which in turn decreases the electrostatic attraction between the cell surface and Na^+^. This reduction in attraction creates a high probability of a mechanistic hindrance to ion uptake.

The functional annotation tool, DAVID (https://david.ncifcrf.gov/), was used to identify the biological processes and functions enriched in *H. congolense* WG10 planktonic cells growing under high salinity compared to the optimum (Fig. 1B). For this, the effect size threshold was set to FC > 1.0, increasing the number of upregulated proteins to 111 from 87 (FC > 1.5). Chorismate and aromatic amino acid (AAA) biosynthesis were the predominantly enriched biological processes. When stressed by hypersalinity, many bacteria are known to accumulate osmoprotectants including sugars and amino acids. These are solutes that do not interfere with cellular metabolism, hence can be concentrated in very large amounts to maintain intracellular-extracellular isotonic balance, i.e. function as salt antagonists. They also stabilize and protect sensitive cellular macromolecules, such as DNA and proteins, against ionic damage [32]. *Halanaerobium* may accumulate osmolytes such as choline, proline, and glutamine during exposure to high salinity in shale reservoirs [16]. GB, a derivative of glycine, might also be accumulated; however, the existence of an intracellular degradation mechanism puts its osomoprotective utility in doubt [16]. Other persistent shale taxa, such as *Candidatus* Uticabacter and *Geotoga*, seem much more likely to use GB as an osmoprotecting compatible solute [16].

**Figure 2:**
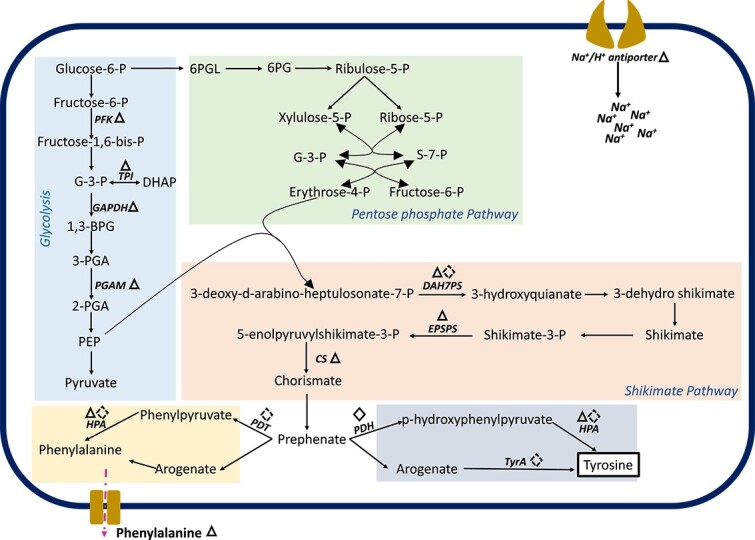
Sodium-proton antiporter and tyrosine biosynthesis are upregulated in *H. congolense* WG10 growing under hypersalinity. Schematic of reaction steps in tyrosine biosynthesis upregulated in *H. congolense* WG10 growing under high (20% NaCl) salinity relative to the optimum (13%). Significantly (*P* < .05) upregulated proteins (enzymes) are bolded and italicized. Triangles and diamonds indicate upregulation in planktonic and biofilm cultures, respectively. Solid shape line denotes FC > 1.0 while dotted shape line denotes 0 < FC < 1.0. *PFK*, phosphofructokinase; *TPI*, triosephosphate isomerase; *GAPDH*, glyceraldehyde 3-phosphate dehydrogenase; *PGAM*, phosphoglycerate mutase; *DAH7PS,* 3-deoxy-d-arabino-heptulosonate 7-phosphate synthase; *EPSPS*, 5-enolpyruvylshikimate-3-phosphate synthase; *CS*, chorismate synthase; *PDH*, prephenate dehydrogenase; *PDT,* prephenate dehydratase; *HPA*, histidinol-phosphate aminotransferase; *TYRA*, arogenate dehydrogenase.

Here, we did not find any proteomic evidence for intracellular choline, proline, glutamine, and GB accumulation in *H. congolense* WG10 growing under high salinity. GB can be excluded from the de novo synthesis criterion as *Halanaerobium* lacks the ability to make it from either glycine or choline [16]. Based on exometabolomic evidence, we discovered that extracellular levels of choline increased under 20% salinity (Fig. 1C), which contrasts what we had expected if choline were a significant osmoprotectant. It is important to note that we intentionally did not provide any of these solutes in the growth medium to assess the inherent ability of *H. congolense* to cope with hypersalinity. As such, it is not surprising that similar to their anabolic enzymes, there was no upregulation of transporters exclusively responsible for the uptake of these solutes. *H. congolense* WG10 potentially handled high salinity by overproducing and intracellularly accumulating tyrosine, in complementarity with the salt-in approach. This is based on two specific lines of evidence—upregulation of several enzymes catalyzing tyrosine biosynthesis and non-detection of tyrosine in the exometabolome (Fig. 1C, Fig. 2, and SI Appendix text). This is surprising as tyrosine is not known for osmoprotection in Bacteria; however, it has been implicated in natural drought and salinity tolerance in plants [33]. Pathways for tyrosine catabolism have been previously identified in three bacterial taxa including *Clostridium* [34], *Pseudomonas* [35], and *Arthrobacter* [36]; however, none of the enzymes exclusively involved in these pathways were found to be upregulated in *H. congolense* WG10 under high salinity. This further demonstrates tyrosine’s potential role in this bacterium as an osomoprotective solute. Additional studies are needed to corroborate this finding.

### Aromatic amino acid synthesis is enriched during biofilm formation in *H. Congolense* WG10

**Figure 3:**
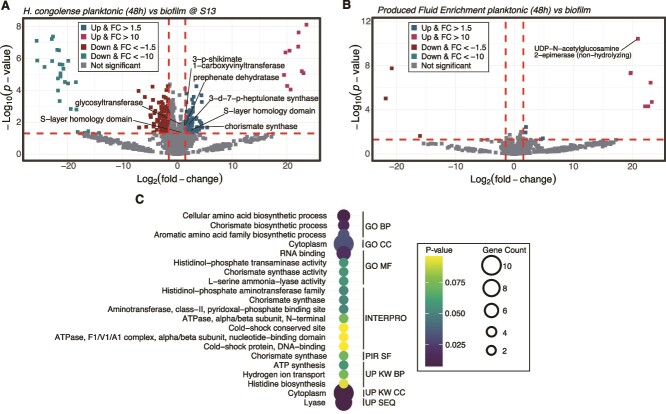
Proteomic changes occurring in *H. congolense* WG10 and PFE mixed microbial consortia during biofilm formation*.* (A) Volcano plot showing the *P*-value and log2 FC of proteins in *H. congolense* WG10 growing under 13% NaCl in planktonic (HRT: 48 h) vs. biofilm mode (48 h incubation). The direction of comparison is planktonic to biofilm. (B) Volcano plot showing the *P*-value and log2 FC of proteins in mixed microbial consortia enriched from shale-produced fluids in planktonic (HRT: 48 h) vs. biofilm mode. The direction of comparison is planktonic to biofilm. In the volcano plots, the horizontal line indicates the threshold of statistical significance, *P* < .05, while both vertical lines delineate |FC| > 1.5. (C) Functional enrichment of proteins that significantly increased (FC > 1; *P* < .05) in *H. congolense* WG10 during biofilm formation. Bubble size indicates gene count. GO BP, gene ontology biological process; GO CC, gene ontology cellular compartment; GO MF, gene ontology molecular function; PIR SF, protein information resource superfamily; UP KW BP, UniProt keywords biological process; UP KW CC, UniProt keywords cellular compartment; UP KW MF, UniProt keywords molecular function.

To understand the molecular mechanisms underlying biofilm formation in *H. congolense* WG10, we compared the whole cell proteomes of planktonic cells grown under 48 h HRT to biofilm cells incubated for 48 h. Both culture types were cultivated under the optimal salinity (13% NaCl). We found 228 proteins to be significantly discriminant, based on the thresholds *P* < .05 and |log2 FC| > 1.5. Approximately 35% (79) of these DEPs were upregulated (FC > 1.5) in biofilm cells (Fig. 3A). They include a broad range of enzymes and structural proteins that we further evaluated. DEPs in the mixed enrichment cultures growing as biofilm vs. planktonic are shown in Fig. 3B but not discussed further in this section.

Functional enrichment analysis using DAVID revealed that chorismate and AAA biosynthesis were enriched in *H. congolense* WG10 biofilm cells (Fig. 3C), hence important for surface aggregation. Chorismate is a central intermediate in the synthesis of three AAAs: tyrosine, phenylalanine, and tryptophan. The involvement of AAAs in various biofilm physiologies has been demonstrated before [37–39], especially their role as key components of flagellin and flagellar hook proteins [40, 41] which facilitate cellular migration to surfaces. The flagella, in addition, acts as an adhesin, helping consolidate microcolony establishment. Moreover, AAAs such as phenylalanine have been identified to modulate chemoreception [42] and chemoattraction which would help biofilm development [43–45]. Furthermore, AAAs have been suggested to activate the expression of quorum sensing and extracellular polymeric substance (EPS) genes [46]. AAA synthesis is also upregulated in *H. congolense* WG10 biofilms relative to planktonic, under 20% NaCl (SI Appendix, Table S1). More research is needed to fully understand the role of AAA metabolism in biofilm formation in shale reservoirs. This is particularly important as previous studies have shown that adding exogenous AAAs can inhibit biofilm development [47].

### Cell envelope maintenance is vital for biofilm formation in shale microbes

**Figure 4:**
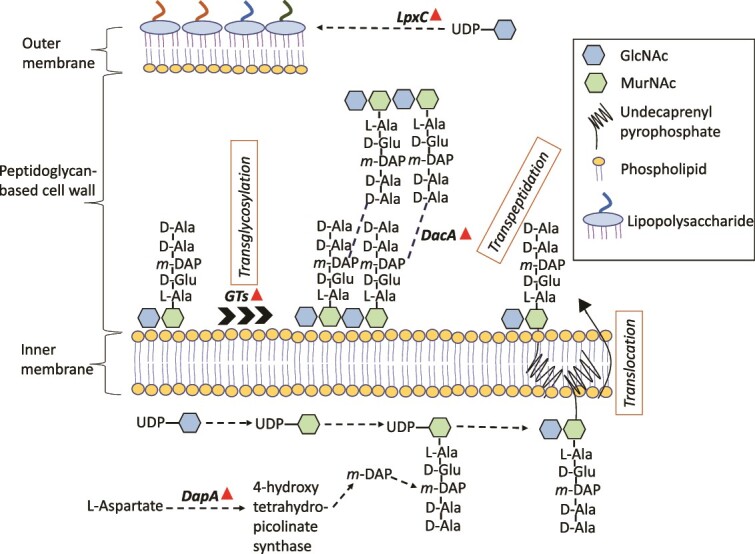
Proteins catalyzing key steps in cell wall and lipopolysaccharide biosynthesis are significantly (*P* < .05) upregulated in *H. congolense* WG10 biofilms relative to planktonic (HRT: 48 h) cells, both of which were incubated under optimal salinity (13% NaCl). Upregulation is denoted by upward-facing triangles. Dotted lines indicate multiple reaction steps. *DapA*, 4-hydroxy-tetrahydropicolinate synthase; *GTs*, glycosyltransferases; *DacA*, D-alanyl-D-alanine carboxypeptidase; *LpxC*, UDP-3-0-acyl-N-acetylglucosamine deacetylase.

A common observation for *H. congolense* WG10 and produced fluid enrichment (PFE) consortia was the upregulation of a few catalytic and structural proteins involved in cell envelope synthesis and maintenance during biofilm growth. For instance, levels of some cell wall biosynthesis enzymes were found to be significantly higher (*P* < .05) in *H. congolense* WG10 biofilms compared to planktonic (HRT: 48 h) cells, both of which were incubated under the optimal salinity (13% NaCl). These include D-alanyl-D-alanine carboxypeptidase (FC = 1.03), 4-hydroxy-tetrahydrodipicolinate synthase (FC = 0.5), and glycosyltransferases (FC = 1.39). The reactions they catalyze are shown in Fig. 4. Other proteins that play various roles in cell envelope integrity and function were also upregulated during biofilm formation in *H. congolense* WG10. These include UDP-3-0-acyl-N-acetylglycosamine deacetylase (FC = 0.6) which catalyzes the biosynthesis of Lipid A, a key component of the outer membrane lipopolysaccharide, and surface (S)-layer homology domains (FC = 2.4) which are modules that display proteins (e.g. S-layer glycoproteins) on the cell envelope. Biomarker analysis using the multivariate receiver operator characteristic curve approach showed S-layer homology domain-containing protein to be a likely indicator of biofilm formation in *H. congolense* WG10 based on a 15-feature random forest model (SI Appendix, Fig. S2B). Similar observations were made for the mixed microbial consortia enriched from produced fluids in biofilm mode vs. planktonic (HRT: 72 h). Here, proteins found in significantly (*P* > .05) higher abundance in biofilm cells include the S-layer homology domain-containing protein (FC = 0.9), glycoside hydrolases (FC = 1.0)—which facilitate cell wall remodeling—, and outer membrane beta-barrel protein (FC = 2.4). Furthermore, cell envelope-associated processes including taxis, communication, signaling, and response to stimulus were enriched in *H. congolense* WG10 biofilm relative to planktonic, under 20% NaCl (SI Appendix, Table S1).

The cell wall’s role in providing structural integrity and protection against physicochemical disturbances is well understood in biological organisms; its role in bacterial biofilm formation not as much. A few studies, however, are beginning to shed some light on this. Cell envelope chemistry likely affects the composition and anchoring of the EPS matrix to surfaces and cell-to-cell adhesion [48]. Furthermore, the bacterial cell wall localizes several proteins with possible critical roles in biofilm formation [49]. In addition, cell wall defects could trigger generalized cellular stress response which might inhibit biofilm formation [50]. We, therefore, propose that in addition to other pertinent adjustments, shale microbes enhance the integrity and robustness of their cell envelopes during biofilm formation.

### Metabolic stress response in shale microbes is mediated by amino acid metabolism and active transport regulation

FPW are collected together with natural gas from shale reservoirs after hydraulic fracturing. Initially, the rate of FPW recovery declines steeply as soon as production commences. This decline continues over time, but it becomes less pronounced. Moreover, the flow rates of wells are intentionally adjusted based on energy demand and other technical factors [51]. Reservoir biogeochemistry varies with flow rate fluctuations mainly due to changes in hydromechanics and nutrient flux, which affect microbial physiologies. We simulated this phenomenon in the laboratory by incubating *H. congolense* WG10 and enriching mixed consortia in shale-produced fluids using a chemostat (continuous culture system) operated under varying HRTs. Within the optimal HRT range (when specific growth rate is at its maximum), exponential growth is prolonged due to the optimal cell to nutrient ratio. Under abnormally high HRT, it is typical for nutrients (including essential carbon and nitrogen) to become limited relative to cell density and for toxic metabolic byproducts to accumulate, and both of these effects create intense metabolic stress. Similar conditions are created in continuously producing shale reservoirs months to years after completion due to drastic reduction in flow rate (higher fluid/microbe residence time).

**Figure 5:**
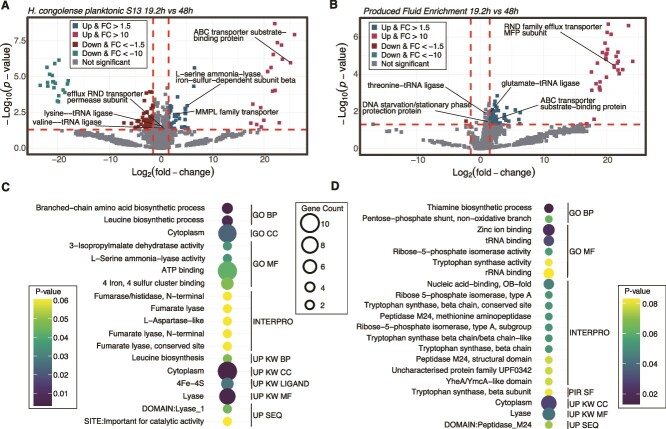
Proteomic changes in *H. congolense* WG10 and PFE consortia grown under high HRT (48 h) vs. low (19.2 h). (A, B) Volcano plots showing the *P*-value and log2 FC of proteins in *H. congolense* WG10 (A) and PFE (B) planktonic cultures, grown under 19.2 h vs. 48 h HRT. The direction of comparison is 19.2 to 48 h. *Halanaerobium congolense* cultures were incubated at optimal salinity (13% NaCl). The horizontal line indicates the threshold of statistical significance, *P* < 0.05, while both vertical lines delineate |FC| > 1.5. (C, D) Functional enrichment of proteins that significantly increased (FC > 1; *P* < .05) in *H. congolense* WG10 (C) and PFE microbial consortia (D), during growth under 48 h HRT relative to 19.2 h. Bubble size indicates gene count. GO BP, gene ontology biological process; GO CC, gene ontology cellular compartment; GO MF, gene ontology molecular function; PIR SF, protein information resource superfamily; UP KW BP, UniProt keywords biological process; UP KW CC, UniProt keywords cellular compartment; UP KW MF, UniProt keywords molecular function; UP SEQ, UniProt sequences.

We sought to investigate the biochemical responses of shale taxa to increasing medium retention time or reduced reservoir flow rate. First, we compared the whole cell proteome and exometabolome of planktonic *H. congolense* WG10 cells incubated at optimum salinity (13% NaCl) under low (19.2 h) vs. high (48 h) HRT (Fig. 5A, and SI Appendix, Fig. S3A). A similar comparison was made for PFE consortia incubated under 19.2 h vs. 48 h HRT (Fig. 5B, and SI Appendix, Fig. S3B). Results of functional enrichment analysis using upregulated proteins under the higher HRT are shown in Fig. 5C and D for *H. congolense* WG10 and the mixed consortia, respectively. We should note that glucose was depleted under the higher HRT in only the PFE reactor, and not the isolate culture. However, other nutrients such as nitrogen and phosphorus might be depleted in both systems under significantly reduced flow rates, constituting starvation stress to cells in addition to metabolic waste toxicity. DAVID functional enrichment analysis was performed using proteins significantly upregulated (FC > 1; *P* < .05) under the higher HRT. Our results revealed an expected upregulation of amino acid metabolism in both the pure and mixed cultures. Enriched biological processes and molecular functions in metabolically stressed *H. congolense* WG10 included branched chain amino acid biosynthesis, leucine biosynthesis, and L-serine ammonia-lyase activity (Fig. 5C). Similarly, enriched functions in the mixed consortia included tryptophan synthase and methionine aminopeptidase activities (Fig. 5D).

We believe that the metabolic stress induced by very long residence times (HRT) in the bioreactor causes shale microbes to ramp up amino acid synthesis, which might be largely directed toward making stress response proteins. In both the isolate culture and mixed consortia, several enzymes involved in amino acid synthesis were upregulated under 48 h HRT relative to 19.2 h. Additionally, in *H. congolense* WG10, there was a significant increase in the abundance of many amino acid-tRNA ligases including lysine, valine, glycine, and asparagine, which are vital for protein synthesis. Similarly, tRNA ligases for glutamate, threonine, and phenylalanine were upregulated in the mixed microbial consortia under the higher HRT. Stress proteins play key roles in bacterial survival during metabolic stress, mainly to maintain the cell. These include averting protein misfolding, breaking down misfolded/damaged proteins, and DNA repair [52, 53]. Although we surprisingly did not observe a significant increase in any stress protein in cells under 48 h HRT relative to 19.2 h, which may be due to the analytical limitations of mass spectrometry-based metaproteomics (extraction bias, ionization inefficiency, posttranslational modification and other chemistry-based biases, abundance threshold, incomplete reference databases, etc.). It is also possible that regulation of amino acid metabolism in shale microbes under metabolic stress might function to reset their energy strategy (SI Appendix text).

Furthermore, we believe that an increase in amino acid metabolism for metabolically stressed shale microbes is tied to the regulation (synthesis and recycling) of membrane-bound transporters, which our findings showed. For both *H. congolense* WG10 and the mixed consortia grown under variable HRTs, we observed unsystematic control of transmembrane active transporters (SI Appendix, Tables S2 and S3). ABC transporters were the most implicated. Several transporters were enriched, and others suppressed under 48 h HRT relative to 19.2 h. Cells facing metabolic stress in the lab bioreactor or shale reservoir must contend with a dilemma. With potential nutrient depletion and accumulation of toxic metabolic byproducts such as organic acids and ions, it is logical that these cells partially shut down their transmembrane transport system or at least make it more stringently selective. This reduces the energy cost of maintaining redundant transporters and limits the entry of toxic metabolites and ions into the cell. In conflict with this energy efficiency goal is the ever present need to scavenge nutrients to remain viable and support essential metabolism. Cells must strike a balance between both necessities. The mixed regulation of transmembrane transport observed in these taxa under different HRTs might also be explained by the fact that a chemostat, despite being operated under continuous stirring, is not a perfect homogeneous system, meaning biochemical responses would most likely differ from cell to cell depending on microenvironment, versus being communally in sync.

We did observe a common trend under the higher HRT for both *H. congolense* WG10 and the mixed consortia-upregulation of efflux proteins that function to rid the cell of unwanted/toxic substances. These include subunits of the efflux RND (Resistance, Nodulation and cell Division) transporter permease and a cation-transporting P-type ATPase (SI Appendix, Tables S2 and S3). Peculiar to *H. congolense* WG10 growing under 48 h HRT vs. 19.2 h, was the upregulation of a Mycobacterial membrane protein Large transporter known to mediate lipid export and toxin extrusion [54]. Increased secretion of unwanted, potentially toxic metabolic byproducts such as acetate by shale bacteria in response to metabolic stress, which we observed in this study (SI Appendix, Fig. S3), might aggravate the risk of biocorrosion posed by the organic acid in the reservoir.

### Extending laboratory-generated hypotheses to field scale

We attempted to validate hypotheses generated from laboratory microcosms at a field scale by analyzing filtered produced fluids sampled at four time points (December 2019, July 2020, December 2020, and May 2021) from a fractured shale well. The first sample was taken approximately a month post-well completion. By the second sample collection date, produced fluid volumes had dropped by over 90% (SI Appendix, Fig. S4). This further concentrates the reservoir brine, upsets the biomass-nutrient balance, and creates conditions for microbial sessility and biofilm formation. The cell-free filtrates were used for exometabolomics while the residues (containing cell pellets) were used for metaproteomics. Unfortunately, due to analytical complications, we were unable to obtain actual protein abundance estimates beyond peptide spectrum matches.

Therefore, we simply searched the entire filter dataset for the proteins we inferred from lab experiments to modulate osmoprotection, metabolic stress tolerance, and biofilm formation strategies. We found several of these proteins in the filter metaproteomes. This includes the Na^+^/H^+^ antiporter and enzymes involved in the chorismate to tyrosine biosynthetic tract. Furthermore, we found proteins that modulate cell wall maintenance and efflux RND transporter permeases, which we showed to be important for biofilm formation and metabolic stress tolerance, respectively (SI Appendix, Dataset 1).

On the other hand, we explored the cell-free produced fluid exometabolome, which were able to detect and quantify using NMR spectroscopy. First, we noticed that concentrations of choline and acetate progressively declined over time (SI Appendix, Fig. S5). This suggests continuous cellular choline uptake for likely use as an osmoprotectant or as a carbon and energy source. We didn’t detect tyrosine and phenylalanine unlike in the lab setup despite metaproteomic indications of their biosynthesis. Hence, compared to the relatively favorable bioreactor conditions where we found evidence for only tyrosine accumulation, *in situ* fractured shale microbiome facing multitiered hostility most likely relies on a vast array of solutes for osmoprotection, including phenylalanine and choline. Our lab experiments showed an increase in extracellular acetate concentrations under lower flow rates. However, in contrast, we observed a temporal decline in acetate levels in shale-produced fluids over time, which suggests microbial reutilization as a carbon and energy source or environmental degradation.

## Findings can inform shale reservoir microbial controls

**Figure 6:**
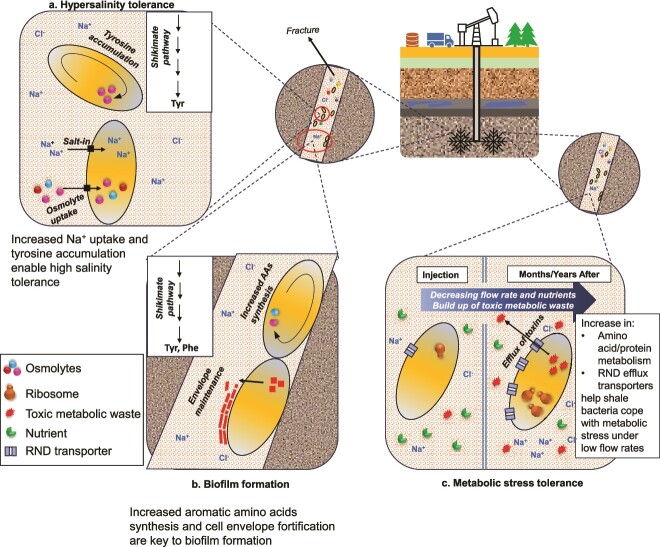
Proposed model for key physiological mechanisms underlying microbial persistence in fractured shale. (A) Hypersalinity tolerance mechanisms include de novo AAAs (tyrosine) accumulation, and uptake of salts (salt-in) and osmolytes (compatible solutes) from the cell’s surroundings. (B) Increase in AAA synthesis and cell envelope maintenance are vital for biofilm formation. (C) Facing metabolic stress, cells ramp up amino acid/protein metabolism and express more RND transporters to extrude toxic metabolic waste.

Attempts to control microbial activities in fractured shale using biocides have often not been very successful as some taxa persist in the reservoir months to years into production. How these organisms survive this deep terrestrial environment has remained largely elusive. Our surprising observation of tyrosine accumulation (Fig. 6A and B), which to the best of our knowledge is unprecedented in bacteria, premises our position that persistent shale bacteria have incredible adaptive flexibility. It is very likely they are capable of utilizing a wide range of other compounds, beyond tyrosine or even AAAs, for osmoprotection and switch between these options depending on the prevailing circumstances. We therefore encourage future mechanistic studies using laboratory reactors and more representative mesocosms to unravel the full extent of adaptive mechanisms in persistent shale bacteria. This is crucial for better management of shale microbiology. In addition, beyond shale reservoirs, we believe bacteria in general might have the ability to use a wide variety of other molecules as osmoprotectants in addition to the commonly known ones including ectoine, GB, choline, and trehalose. It is important to note that additional studies, including genetic experiments, are required to confirm tyrosine accumulation and its proposed role in hypersalinity tolerance in fractured shale bacteria. For one, tyrosine accumulation under high salinity could be for reasons other than osmoprotection, such as carbon storage.

This study additionally implicates amino acid metabolism as a key mechanism in osmotolerance, biofilm formation, and metabolic stress response in shale microbes. An earlier study [16] elucidated the centrality of amino acids to microbial life in fractured reservoirs. GB, an amino acid derivative, is used as an osmoprotectant as well as an energy source by most taxa. GB fermentation to TMA, the first step in a co-feeding network that produces biogenic methane, uses amino acids as reductants (Stickland fermentation). Additionally, Ca. Uticabacter uses glycine to reduce sarcosine to monomethylamine, another methanogenic substrate. Our study extends the role of amino acids, especially the aromatic species, as facilitators of microbial growth and maintenance in subsurface shale (Fig. 6C). Tyrosine is accumulated under hypersalinity and AAA synthesis is upregulated during biofilm formation and under metabolic stress resulting from very low chemostat flow rates. Therefore, sulfur-containing biocides like chloromethylisothiazolinone (CMIT, or MCI, 5-chloro-2-methyl-3(2*H*)-isothiazolinone) and methylisothiazolinone (MIT, or MI, 2-methyl-3(2*H*)-isothiazolinone), which inhibit cellular metabolism by reacting with a wide range of amino acids [55], should be reevaluated for biocontrol use. Compared to glutaraldehyde (27%) and dibromo-nitrilopropionamide (24%), CMIT and MIT are only used in about 1.1% of engineered shale [55]. Compounds that specifically disrupt AAA structures or inhibit their metabolism could prove to be promising biofouling control agents. In addition, we recommend further studies to understand the efficacy or lack thereof of glutaraldehyde and other commonly used biocides against biofilm growth in shale bacteria and decipher their underlying mechanisms of action if efficacious.

In conclusion, we have demonstrated how model bacterium, *H. congolense* WG10, and mixed microbial consortia enriched from shale-produced fluids adapt to hypersalinity and metabolic stresses, by integrating metaproteomics and exometabolomics. The use of laboratory microcosms made it possible to minimize confounding factors to elucidate these complex biochemistries. We show evidence that tyrosine is accumulated under high salinity, and that amino acids might play a key role in biofilm formation and metabolic stress response. This study therefore extends our knowledge of life in subsurface energy systems, especially in high salinity shale reservoirs, to inform improved microbiome control and manipulation approaches to enhance the efficiency of energy extraction.

## Supplementary Material

Supplementary_ISME_Comm_Revised_ycae149

## Data Availability

The original contributions presented in the study are included in the main article and supplementary materials. All mass spectrometry proteomics data were deposited in NEXUS (Project Number: 51545), the public user portal for Environmental Molecular Sciences Laboratory (EMSL), PacificNorthwest National Laboratory (PNNL), and are available at https://dx.doi.org/10.25582/data.2023-11.2949061/2216895. Further inquiries can be directed to the corresponding author.
