# Supplementary material for: Aromatic amino acid metabolism and active transport regulation are implicated in microbial persistence in fractured shale reservoirs
Source: ISME Commun. 2024 Nov 26;4(1):ycae149. doi: 10.1093/ismeco/ycae149 (PMC11637423; doi:10.1093/ismeco/ycae149)
Supplement: Supplementary_ISME_Comm_Revised_ycae149 [file supplementary_isme_comm_revised_ycae149.docx]

**Aromatic amino acid metabolism and active transport regulation are implicated in microbial persistence in fractured shale reservoirs**

Chika Jude Ugwuodo^1,2^, Fabrizio Colosimo^3^, Jishnu Adhikari^4^, Samuel O. Purvine^5^, Elizabeth K. Eder^5^, David W. Hoyt^5^, Stephanie A. Wright^5^, Mary S. Lipton^5^, and Paula J. Mouser^2*^

^1^Natural Resources and Earth Systems Science, University of New Hampshire, Durham, NH, United States.
^2^Department of Civil and Environmental Engineering, University of New Hampshire, Durham, NH, United States.
^3^New England Biolabs, Ipswich, MA, United States.
^4^Tetra Tech Inc., King of Prussia, PA, United States.
^5^Biological Sciences Division, Pacific Northwest National Laboratory, Richland, WA, United States.

***Correspondence:**Paula J. Mouser
([Paula.Mouser@unh.edu](mailto:Paula.Mouser@unh.edu))

**Supplementary Information**

*Tyrosine accumulation in H. congolense WG10 under high salinity*

In cells, aromatic amino acid synthesis typically proceeds through the shikimate pathway whose end product is chorismate. Enzymes catalyzing the steps of the shikimate pathway, which begins with the condensation of phosphoenolpyruvate with erythrose 4-phosphate and ends in chorismate synthesis include: 3-deoxy-d-arabino-heptulosonate-7-phosphoate synthase, 3-dehydroquianate synthase, 3-dehydroquianate dehydratase, shikimate 5-dehydrogenase, shikimate kinase, 5-enolypyruvylshikimate 3-phosphate synthase and chorismate synthase. Tyrosine, phenylalanine and tryptophan are synthesized from chorismate via different pathways. For tyrosine, chorismate mutates to prephenate which is then converted to either arogenate or p-hydroxyphenylpyruvate. Arogenate is dehydrogenated while p-hydroxyphenylpyruvate acquires an amino group to form tyrosine.

We observed that in *H. congolense* WG10 planktonic cells growing under high (20%) salinity vs. the optimum (13%), many enzymes catalyzing reactions in the shikimate pathway were upregulated (FC > 1.0; *P* < 0.05), including: 3-deoxy-D-arabinoheptulosonate-7-phosphate synthase, 5-enolypyruvylshikimate 3-phosphate synthase and chorismate synthase. Abundance of chorismate mutase, which converts chorismate to prephenate, didn’t increase at high salinity. However, prephenate dehydrogenase/arogenate dehydrogenase family protein was moderately (FC = 1.13) upregulated in *H. Congolense* WG10 biofilm cells under high salinity but not in their planktonic counterpart. Prephenate dehydrogenase produces p-hydroxyphenlypyruvate from prephenate, which is then converted to tyrosine by the action of an aromatic amino acid aminotransferase. We believe histidinol-phosphate transaminase, which was significantly elevated in the planktonic cells (FC > 3) and marginally upregulated in the biofilm (FC = 0.82), catalyzes this transamination reaction (Fernandez et al., 2004). On the other hand, arogenate dehydrogenase catalyzes the direct conversion of arogenate to tyrosine.

In addition to tyrosine, the other two aromatic amino acids in bacteria – phenylalanine and tryptophan – can also be synthesized from chorismate. We think prephenate dehydratase, although only marginally upregulated (FC = 0.66) in *H. Congolense* WG10 biofilm cells but not in their planktonic counterpart, forms phenylpyruvate from prephenate. Histidinol-phosphate transaminase then converts phenylpyruvate to phenylalanine. No enzyme involved in conventional tryptophan synthesis from chorismate was significantly increased under high salinity. *H. Congolense* WG10 planktonic cells, however, appeared to only accumulate tyrosine and not phenylalanine under high salinity, evident in the increased levels of phenylalanine in the medium. Tyrosine wasn’t detected in the exometabolome. Also, no enzyme involved in tyrosine catabolism was found in the cellular proteome. Both observations lend strong support to our claim that tyrosine is accumulated as an osmoprotectant in *H. Congolense* WG10 and helps it cope with high salinity stress.

To sustain the upregulation of the shikimate and tyrosine biosynthetic pathways under high salinity stress, *H. Congolense* WG10 must ensure sufficient supply of the starting metabolites, phosphoenolpyruvate (PEP) and erythrose-4-phosphate (E4P). While PEP is a glycolytic intermediate, E4P is mainly synthesized via the pentose phosphate pathway (PPP). We found that many glycolytic enzymes were upregulated in *H. Congolense* WG10 benthic and planktonic cells growing under high salinity (20%) compared to the optimum (13%). These include glyceraldehyde-3-phosphate dehydrogenase, triose phosphate isomerase, phosphoglycerate mutase and 6-phosphofructokinase. Curiously, there was no proteomic evidence for the upregulation of the PPP pathway. We attribute this to either the analytical limitations of mass spec-based global proteomics, or alternative sources of E4P in *H. Congolense* WG10. It is crucial to note that upregulation of glycolysis also plausibly serves to meet the high energy demand of osmoadaptation.

*Biomarker analysis of H. congolense WG10 growth under optimal (13% NaCl) vs. high salinity (20% NaCl)*

We performed a multivariate receiver operator characteristic curve (ROC) based exploratory analysis using the Biomarker Analysis module of MetaboAnalyst v5.0 to predictively model the differences in the proteomes of *Halanaerobium congolense* WG10 growing under 20% vs 13% NaCl. For this purpose, data from planktonic and biofilm cells were combined and categorized based on salinity. A panel of 15 biomarker proteins (***SI Appendix*, Figure S1B)** resulted in an area under the curve (AUC) value of 0.9 (***SI Appendix*, Figure 1C)**. Out of these, 7 were upregulated under high salinity vs. the optimum and could be useful as indicators of hypersalinity tolerance potential in subsurface reservoir microbiomes as well as novel biocidal targets.


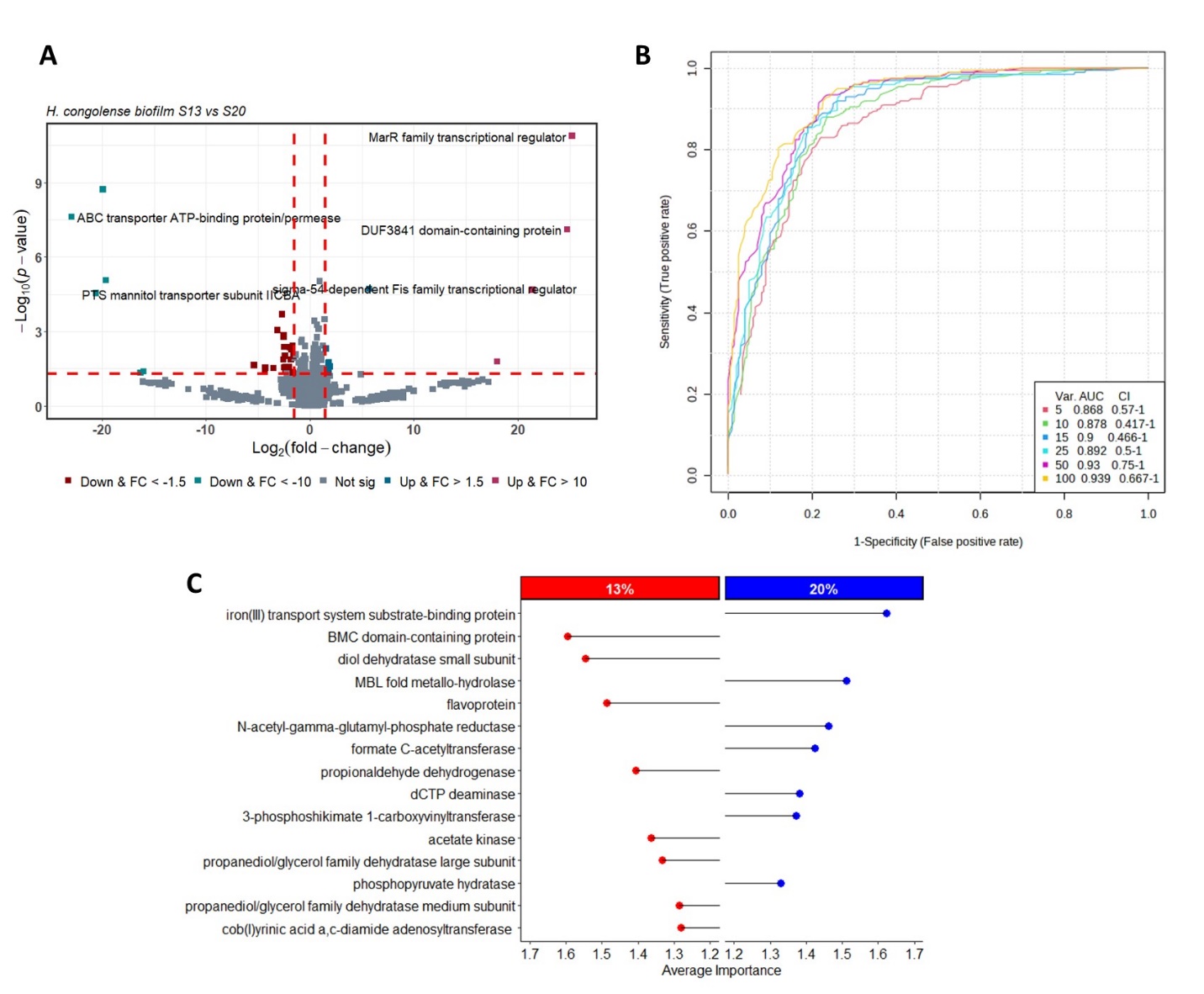


**Figure S1. Proteomic changes in *H. congolense* WG10 grown in biofilms under 13% (optimum) vs. 20% (high) NaCl. A.** Volcano plot showing the *P*-value and log2fold change (FC) of proteins in *H. congolense* WG10 biofilm cells grown at 24 h HRT under 13% vs. 20% NaCl. The direction of comparison is 13% to 20%. The red horizontal line indicates the threshold of statistical significance, *P* < 0.05, while both red vertical lines delineate |FC| > 1.5. **B.** Receiver operating characteristic (ROC) curves based on cross validation performance of Random Forest models of variations in the proteomic profiles of *H. congolense* WG10 planktons and biofilms grown under 13% vs. 20% NaCl, using different number of features (proteins). **C.** Random forests (RF) classification performance of the 15 proteins most discriminant (ranked by mean importance measure) of *H. congolense* WG10 planktonic and biofilm cells grown under 13% vs. 20% NaCl. Red and blue denote higher abundance at 13% and 20%, respectively.


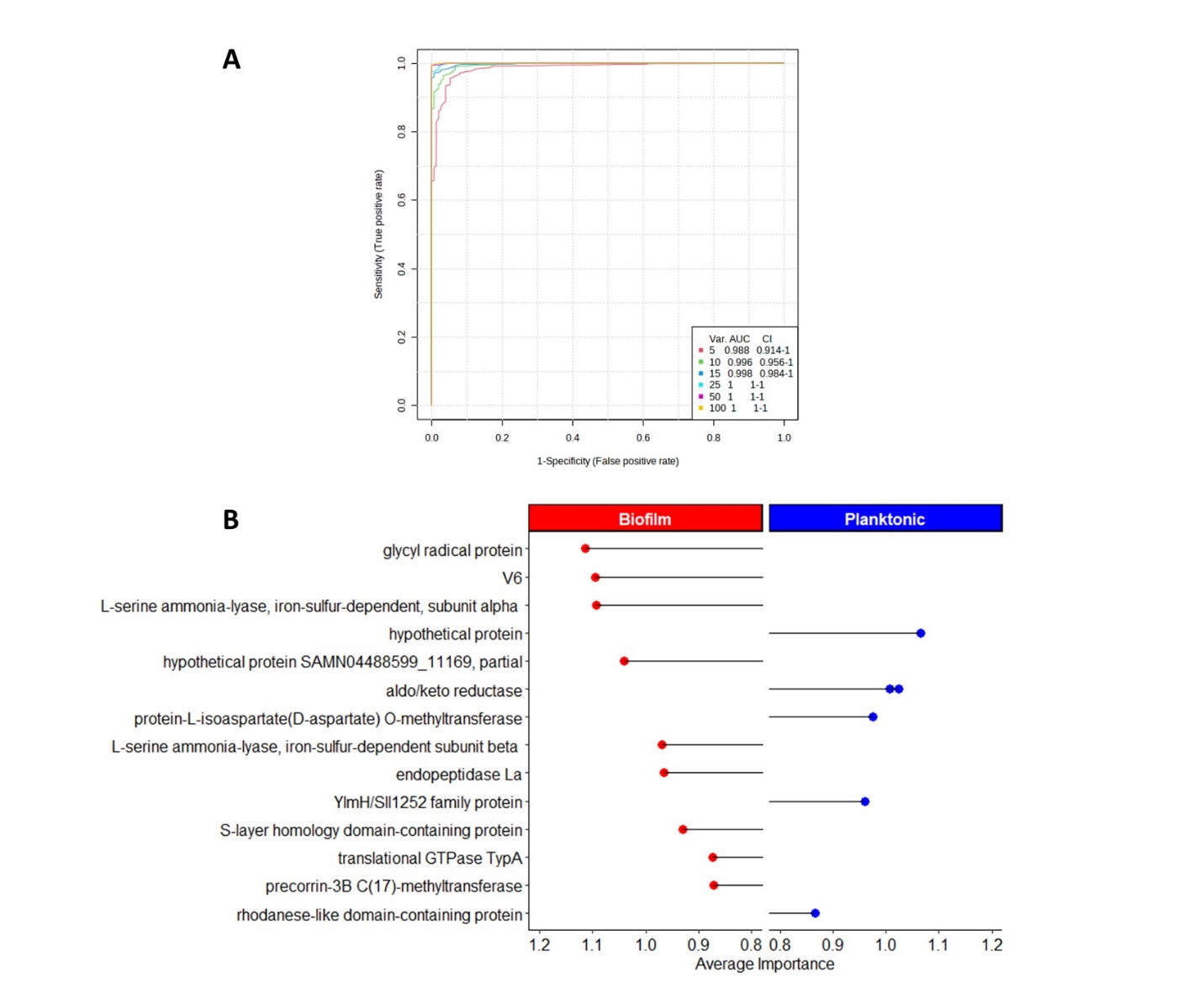


**Figure S2. A.** Receiver operating characteristic (ROC) curves based on cross validation performance of Random Forest models of variations in the proteomic profile of *H. congolense* WG10 during biofilm growth vs. planktonic under 13% NaCl, using different number of features (proteins). **B.** Random forests (RF) classification performance (mean importance measure) of the 15 proteins most discriminant of H. congolense WG10 growing under 13% NaCl in planktonic (48 h HRT) vs. biofilm mode. Red and blue denote higher abundance in biofilm and planktonic cells, respectively.

*Upregulation of amino acid metabolism in shale microbes under lower flow rates might be linked to changes in energy strategy.*

A substantial portion of the enriched amino acid biosynthetic activity found in *H. Congolense* and the mixed microbial consortia under the higher HRT could be geared towards making new enzymes needed to switch to alternative carbon catabolism networks. This is critical in instances where the preferred carbon substrate is in low supply. On the other hand, some amino acids might be funneled into carbon catabolic pathways for energy production. Despite no compelling evidence, the enrichment of L-serine ammonia lyase in starving *H. Congolense* WG10, for instance, makes this a possibility. This enzyme catalyzes the conversion of L-serine and L-threonine to pyruvate and α-ketobutyrate, respectively (44). Pyruvate could then be converted to lactate, formate, acetate, or ethanol to generate ATP and dispose of the reductant, NADH.

**Table S1.** Functions enriched (FC > 1; *p* < 0.05) in *H. congolense* WG10 biofilm vs. planktonic (48 h HRT) growing at 20% NaCl.

| **Go Term - Biological Process** | **Gene Count** | **P-Value** |
| --- | --- | --- |
| chemotaxis | 5 | 0.00075 |
| taxis | 5 | 0.00075 |
| locomotion | 5 | 0.00075 |
| cell communication | 5 | 0.00430 |
| signaling | 5 | 0.00430 |
| signal transduction | 5 | 0.00430 |
| response to external stimulus | 5 | 0.01300 |
| response to chemical | 5 | 0.03700 |
| aromatic amino acid family biosynthetic process | 4 | 0.04500 |
| monosaccharide metabolic process | 4 | 0.04900 |
| aromatic amino acid metabolic process | 4 | 0.05300 |
| pentose-phosphate shunt, non-oxidative branch | 2 | 0.08200 |
| amino acid biosynthetic process | 6 | 0.08700 |
| amino acid metabolic process | 8 | 0.09100 |

**Table S2.** Differentially expressed transporters in *H. Congolense* WG10 grown at optimal salinity (13% NaCl) under 19.2 h versus 48 h hydraulic retention time.

**Table S3.** Differentially expressed transporters in produced fluid enrichment (PFE) consortia incubated under 19.2 h versus 48 h hydraulic retention time.

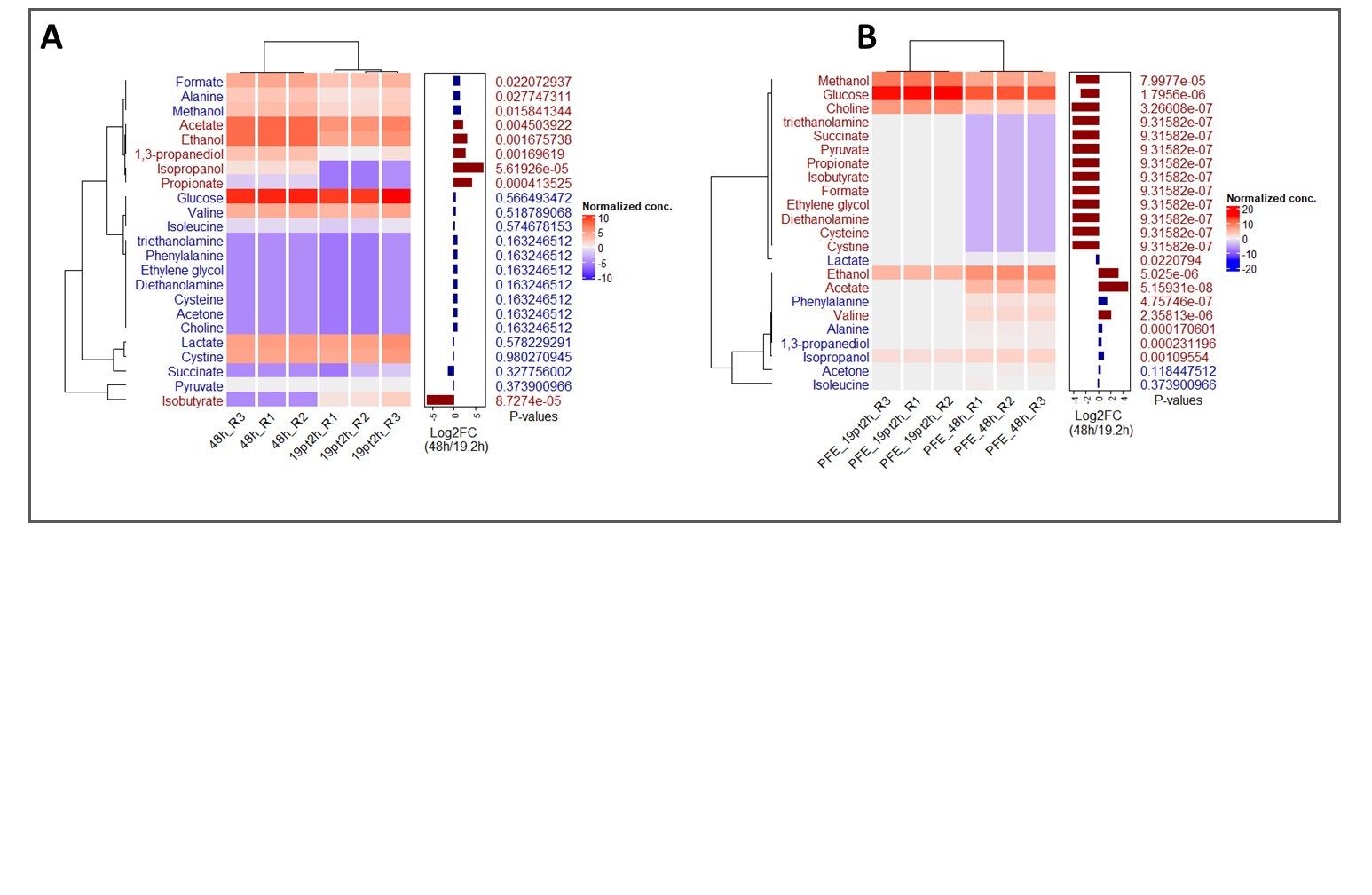


**Figure S3**. **Exometabolomic changes in *H. congolense* WG10 and produced fluid enrichment consortia grown under 19.2 vs. 48 h HRT. A.** Heatmap of normalized and scaled concentrations of extracellular metabolites produced by *H. congolense* WG10 planktonic cells grown at 13% NaCl under 19.2 h vs. 48 h HRT, annotated with a bar plot showing the log2fold changes (FC) and corresponding *P*-values. **B.** Heatmap of normalized and scaled concentrations of extracellular metabolites produced by produced fluid enrichment (PFE) consortia grown under 19.2 h vs. 48 h HRT, annotated with a bar plot showing the log2fold changes (FC) and corresponding *P*-values. Red label color indicates significance (|FC| > 1.5 & *P* < 0.05) while blue denotes non-significance. Rows are clustered by Pearson’s correlation.


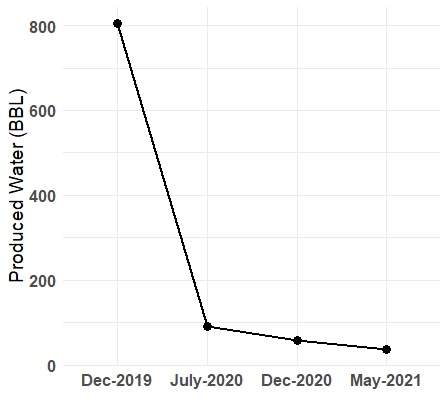


**Figure S4.** Temporal trend of flowback and produced water volumes recovered from the fractured shale well.


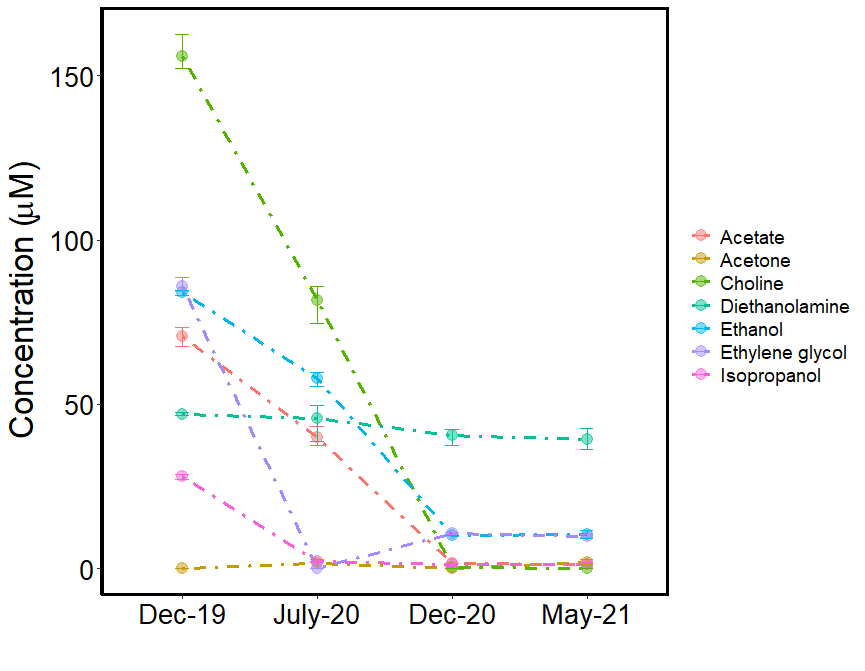


**Figure S5.** Variations in levels of metabolites detected in cell-free produced fluids sampled from the fractured shale well across four timepoints.
